# Be SMART: examining the experience of implementing the NHS Health Check in UK primary care

**DOI:** 10.1186/s12875-014-0212-7

**Published:** 2015-01-22

**Authors:** Rachel L Shaw, Helen M Pattison, Carol Holland, Richard Cooke

**Affiliations:** School of Life & Health Sciences, Aston University, Birmingham, B4 7ET UK

**Keywords:** Cardiovascular diseases, Public health, Preventive medicine, Health behaviour, Intervention studies, Qualitative research

## Abstract

**Background:**

The NHS Health Check was designed by UK Department of Health to address increased prevalence of cardiovascular disease by identifying risk levels and facilitating behaviour change. It constituted biomedical testing, personalised advice and lifestyle support. The objective of the study was to explore Health Care Professionals’ (HCPs) and patients’ experiences of delivering and receiving the NHS Health Check in an inner-city region of England.

**Methods:**

Patients and HCPs in primary care were interviewed using semi-structured schedules. Data were analysed using Thematic Analysis.

**Results:**

Four themes were identified. Firstly, *Health Check as a test of ‘roadworthiness’ for people*. The roadworthiness metaphor resonated with some patients but it signified a passive stance toward illness. Some patients described the check as useful in the theme, *Health check as revelatory*. HCPs found visual aids demonstrating levels of salt/fat/sugar in everyday foods and a ‘traffic light’ tape measure helpful in communicating such ‘revelations’ with patients. *Being SMART and following the protocol*revealed that few HCPs used SMART goals and few patients spoke of them. HCPs require training to understand their rationale compared with traditional advice-giving. *The need for further follow-up* revealed disparity in follow-ups and patients were not systematically monitored over time.

**Conclusions:**

HCPs’ training needs to include the use and evidence of the effectiveness of SMART goals in changing health behaviours. The significance of fidelity to protocol needs to be communicated to HCPs and commissioners to ensure consistency. Monitoring and measurement of follow-up, e.g., tracking of referrals, need to be resourced to provide evidence of the success of the NHS Health Check in terms of healthier lifestyles and reduced CVD risk.

## Background

Cardiovascular disease (CVD) is a leading killer due in part to increasing obesity and sedentary lifestyles. NICE’s framework [1] and the UK National Health Service (NHS) Health Check [2] were designed to help prevent CVD by identifying risk of heart disease, stroke, type 2 diabetes and kidney disease. NHS Health Check is a UK national prevention programme developed by the Department of Health [2]. Its aim is to identify cases of CVD and reduce its risk by preventing new cases of CVD and preventing further complications when a diagnosis is made. It involves inviting all patients (aged 40–74) to health check appointments every five years and providing them with a 10 year CVD risk score and personalised management plan. This plan involves *personalised advice* and *lifestyle support*, which were embedded in the programme to help tackle behaviour change (e.g., diet, physical activity, smoking, alcohol consumption). Tests included cholesterol, blood glucose, blood pressure, and body mass index (BMI). Checks are held in UK General Practice (GP) surgeries and in the community and are delivered by General Practitioners (GPs), practice nurses and Health Care Assistants (HCAs). We were commissioned by Heart of Birmingham Teaching Primary Care Trust (HoBtPCT; now subsumed under Public Health England in Birmingham) to examine patients’ and Health Care Professionals’ (HCPs) experiences of how the check was implemented in that region. The NHS Health Check was delivered in the HoBtPCT region by General Practitioners, Practice Nurses, and Health Care Assistants; during the evaluation period (2010–2011), it included the following:

1. Appointment (year 1):
  - Biomedical tests: cholesterol, blood glucose, blood pressure, and body mass index (BMI);
  - Use of visual aids (pots of fat/salt/sugar), traffic light tape measure;
2. Initial follow-up (once blood results received; may involve a second appointment; year 1):
  - 10 year CVD risk score following results of tests;
3. Management programme (at appointment or initial follow-up):
  - SMART goals set to create a personalised plan to change health behaviours;
  - Lifestyle support may be recommended to help with particular behaviours, e.g., smoking cessation, weight loss, physical activity;
4. Follow-up appointment (year 5):
  - Repeat of biomedical tests;
  - Revision of management programme.

Designed by the UK Government Department of Health (DH) as a ‘case-finding’ public health intervention, the NHS Health Check aimed to: detect risk levels (leading to diagnosis), communicate risk to patients, and provide information and support. The manual for HCPs delivering the check included scripts to use with patients, SMART (Specific, Measurable, Achievable, Results-focused, Timely) goals sheets, test results forms, a lifestyle referral map, a food box, and a ‘traffic light’ tape measure. The food box included pots of fat, salt and sugar indicating recommended daily amounts and pots showing the fat/salt/sugar levels in everyday foods, e.g., yoghurt, oven chips, cheese, chocolate, fruit juice.

Creating SMART goals constituted the *personalised advice*. Patients at risk were referred to *lifestyle support* services, e.g., smoking cessation counselling, walking groups, nutritionists, alcohol advice. The central objective was to communicate risk to patients and facilitate health behaviour change. There have been criticisms of the NHS Health Check, questioning its evidence base [3]. Nevertheless, we know goal-setting is successful at changing behaviour and the specificity of making plans in *SMART goals* (or action plans) makes them particularly effective [4-6]. A key challenge is communication between HCPs and patients [7]. A common analogy used to indicate the intended regularity and significance of the check is an ‘MOT’ - a UK motor vehicle check of roadworthiness conducted by the Ministry of Transport - but findings are inconsistent regarding the effectiveness of using this language [8-10].

The framework of this research is evidence based healthcare; its focus is on the context of implementation and acceptability to staff and patients [11]. We were mindful of the theoretical and empirical evidence which has identified predictors and explanations of behaviour change relevant to the design of the NHS Health Check [6]. Thus, our expectation was that using SMART goals to aid *personalised advice* and providing support and feedback through *lifestyle support* would facilitate successful behaviour change. This study aimed to: examine the experience of HCPs delivering the NHS Health Check and fidelity to protocol, i.e., was it delivered as intended [11-13]; explore patients’ experience of the check and *personalised advice* received; and explore HCPs’ and patients’ perceptions of the feasibility of *lifestyle support* for facilitating behaviour change within the context of everyday life.

## Methods

Approval was obtained from Birmingham and Black Country NHS Research Ethics Committee and Research and Development Department to recruit patients, HCPs, GPs, practice managers and other staff involved in recruitment and/or delivery of the NHS Health Check programme in the HoBtPCT region in 2010–11.

Patients and HCPs were recruited from primary care and the community through lead clinicians within HoBtPCT. Eligible patients were identified by practice managers or lead clinicians and asked either face-to-face or on the telephone whether they were happy to be contacted by a researcher. Practitioners, administrators and managers involved in the organisation or delivery of the check were eligible to participate and were asked whether they were happy to be contacted by a researcher by senior staff involved in the delivery of the check in the HoBtPCT region. Once individuals had consented to be contacted, the researcher sent an information sheet and spoke with them on the telephone or in person to explain the study and arrange the interview. Written consent was obtained at the beginning of the interview which offered another opportunity to answer any questions participants had. The HoBtPCT region is an inner city area of Birmingham, the second largest city in England. It has a relatively high black and minority ethnic population and high levels of deprivation compared to the rest of the country.

We recruited 31 staff and 23 patients from HoBtPCT practices (see Tables 1 and 2). Staff included GPs, Practice Nurses, and Health Care Assistants with a range of years’ experience, all of whom received training from HoBtPCT. We also recruited staff involved in inviting patients to attend (see Tables 1 and 2). Semi-structured interviews were conducted by research assistants (YC, MD) and a Health Psychologist (RC), all non-clinicians able to offer a naïve view, helpful in eliciting accounts [14]. Verbatim transcripts were analysed using thematic analysis [15]. Analysis was inductive, i.e., data-driven; RS led analysis of HCPs, MD analysed patients’ accounts; themes by participant group were generated independently and discussed with the research team to reach agreement. Grouped themes were entered into a matrix for cross-case analysis; a combined set of themes is reported with interpretative commentary. Data saturation was achieved; many issues were repeated in later interviews with nothing new arising. Validity was achieved through triangulation: multiple analysts were involved and multiple perspectives on the same events were sought by gathering accounts from patients and a range of HCPs.

**Table 1:** **Staff recruited who were involved in delivering the NHS Health Check**

**Profession**	**Role in NHS health check**	**Frequency**
Practice manager (PM)	Management, administration	6
General practitioner (GP)	Management, delivery	9
Practice nurse (PN)	Delivery	4
Health care assistant (HCA)	Delivery	6
Alterative provider director (APD)	Management, administration	1
Call centre manager (CCM)	Management of invitation and appointment setting for alternative providers	1
Call centre operative (CCO)	Invitation, appointment setting	2
Alternative provider registered practice nurse (APRN)	Delivery	2

**Table 2:** **Demographic details of patients interviewed within 4 weeks of receiving an NHS health check**

**Ethnicity and sex**	**Frequency**
White British, female	5
White British, male	7
White Irish, female	1
White Irish, male	1
Black British, female	1
Black British, male	3
South Asian, female	2
South Asian, male	1
Afghan, male	1
Somali, male	1

## Results

Themes generated in the analysis include: *Health check as a test of ‘roadworthiness’ for people*, *“It’s an eye-opener”: health check as revelatory*, *Being SMART and following the protocol*, and *“I should be monitored more”: the need for further follow-up*. Each theme will be described in turn using data extracts to illustrate their significance for making sense of how the NHS Health Check was experienced by staff and patients.

### Health check as a test of ‘roadworthiness’ for people

As in previous research we found the check had been framed as a test of ‘roadworthiness’ for people or an ‘MOT’ , a test for cars in the UK carried out every three years. For Patient 8 (P8) this metaphor resonated strongly; the check could identify if anything was “wrong” and if so whether it could be fixed. P8 was unaware of the wider goal of prevention and lifestyle change.

*Well I was under the impression the doctor had put me forward just to put my mind at rest but I didn’t realise it was part of a bigger thing. [..] I thought it was just to see if there was anything wrong with me to begin with. (P8)*

For P8 the check was something done in response to the General Practitioner’s (GP) request and was not connected to lifestyle issues or CVD risk. As such P8 adopted a passive stance toward the check; there was no awareness that it may result in lifestyle changes or help prevent ill-health in the future. Patient 11 also displayed a passive approach, lacking in agency by thinking it was an administrative requirement. P11 admitted asking no questions, signifying an unquestioning trust and relinquishment of control to the GP.

*I just thought, you know, that because our GP is gone with another surgery I just thought they want a general check-up or something like that. I didn’t know what it was, I didn’t even ask questions. I didn’t go into it too much why they were doing it. (P11)*

Other patients knew about the type and purpose of tests involved and assumed correctly they had been invited because of their age, for example Patient 15.

*I: Do you know why you were invited for a health check?*

*P15: I would believe because when you come to a certain age in life it’s best to get these things done because certain things can happen to you. So it’s best detected in the early stages. [..]*

*I: Do you know what illnesses or diseases the health check is looking to detect?*

*P15: Um diabetes, um high blood pressure, um high cholesterol um I can’t think of anything else what it could be. I guess if anything else you know might come up with the actual results with the bloods then they would probably tell you.*

However, P15’s suggestion that “certain things can happen to you” denotes a passive tone; it lacks an active voice. Health Care Assistant 5 also suggests that denying agency in elevated risk of lifestyle-related disease is problematic.

*You get your other ones which are just coming in for a bit of an MOT and regardless of what the score is, they still don’t do anything about it. (HCA5)*

Such externalisation of causes of illness frees patients of the responsibility to take action.

### “It’s an eye-opener”: health check as revelatory

This theme explores awareness and readiness to change. HCPs described the food box and ‘traffic light’ tape measure as particularly useful in revealing to patients their risk status, e.g., Practice Nurse 4.

*And the resource box, yes, which we use a lot. We find that a visual image of this is what a bag of Haribos looks like as a bag of sugar is highly effective. Particularly with all multi-languages going on you can just say “look” and wave it at them. Visual resources are good for different languages. (PN4)*

Seeing what they were putting in their bodies when they ate high fat/salt/sugar foods was described as a powerful tool by HCPs. It was also useful because it facilitated information provision and feedback because it helped overcome language barriers. Furthermore, they helped patients see for themselves whether they were overweight and whether they ate foods high in fat/salt/sugar.

Despite revealing their level of risk, several HCPs were sceptical of patients’ ability to change, even if risk was identified. The concern for General Practitioner 2 was that initial changes would not last and that bad habits would return.

*My personal experience has been that in that initial phase the shock is enough to stop them over-eating. Unfortunately, as is human nature, they forget. (GP2)*

The longevity of change required to keep CVD risk low was perceived as substantial and to maintain change, Practice Nurse 1 believed there was a need to be committed to communicating risk and being proactive about demonstrating that risk to patients.

*Well you just do it verbally, you say you know “you’re overweight and the computer’s telling me”.. you sort of say “the computer’s saying you’re two stones overweight and you know, you’re carrying too much fat around” and you’d show them a tape measure and obviously it’s got the green and the orange and the red and you say “you’re in the red zone, that’s no good”. (PN1)*

For some HCPs the stories about patients seemed to merge with their own. Their talk of patients’ rationalisations for not taking on more physical activity seemed to resonate with their own experience. The use of ‘you’ often reveals a hidden ‘I’. For example, Health Care Assistant 3 began describing her patients’ busy-ness in generalised terms but the language became more vivid, suggesting an empathic position which may also fit her own situation.

*I think both because women are working as well and like they say that when they get in they start cooking in the evening, they can’t get out to the gym or whatever. [..] By the evening you are absolutely shattered, like you know you’ve got children, you’ve got your family, you’re doing just all your chores. It’s just so exhausting. (HCA3)*

Delivering the check, especially for those HCPs with less experience, may have been self-revelatory in terms of understanding their own behaviour and their response to it.

Some patients had taken up physical activity (e.g., Patient 15) while others believed change was important but had not yet translated this into behaviour.

*Well the walking I do generally but I started going to Zumba now so I’ve been doing that Mondays and Fridays. That’s an hour each day. And I started doing some sit-ups of a morning. Do ten minutes before, you know, I actually get myself ready for work. So it’s, you know, I think it’s given me like a wake-up call sorta thing to get yourself you know sort of in shape so I think it’s a good thing yes. (P15)*

P10 described this intention to change as a “mild resolution” demonstrating an intention to take small steps to initiate lifestyle change.

*P10: What I could do to change my diet would be to sort of cut out in between meals, snacks, biscuits and things and cut down on that. That I think is a sort of a mild resolution to do that and that’s the main thing.*

*I: So is that something that you thought about and settled on since the appointment?*

*P10: I think it sort of reinforced the feeling I should be doing that.*

Similarly, Patient 11 described the health check as an “eye-opener” but not simply in terms of making lifestyle change, in the sense that embodied the essence of preventative medicine; it made patients aware that there were lifestyle-related diseases to which they may be susceptible and may be able to prevent.

*It’s really good. It makes you aware of what problems are around. What you can get and that. It is really good. It teaches you. If you are going there to listen to or you’re going to take it on board, then it makes you aware of everything. But if you just go, just for a general check and that’s it, it’s nothing for you. But it’s an eye-opener for people who would want to do things properly. (P11)*

### Being SMART and following the protocol

SMART goals were an integral part of the check but we found mixed reports from patients and HCPs about their use. Some patients remembered them but did not set any goals during the check.

*I: Have you heard of SMART goals - specific, measurable, realist, timely?*

*P2: Yes.*

*I: But they didn’t go through setting any goals with this [SMART goals sheet]?*

*P2: No, they didn’t do anything like that.*

For those who had not used the SMART goals as intended, this was disappointing because it indicated only part-implementation of the check which lacked the *personalised advice* component.

*I was given a bit of paper with SMART goals on but it wasn’t really discussed. [..] There was no sort of encouragement but there you go. Maybe that’s just the procedure. (P14)*

Nevertheless, some HCPs did use SMART goals as intended and found them beneficial, e.g., Alternative Provider Registered Nurse 1.

*We usually set goals on the sheet of paper you’ve actually got. There’s usually a section where you can sort of advise and get them to say what they’re happy to do. For example, I always say to them start off with a ten minute walk a day and building up on it. And the fact that you don’t necessarily need to go to a gym to keep fit and well, by using what you’ve got at home. And then on the right hand side [of the sheet] you know you say if they can set their goals with you, or measures, on how they’re going to be able to do it. (APRN1)*

For APRN1, the SMART goals incorporated a number of techniques: a visual aid for patients to take away; they re-framed “keep fit” as something beyond the gym or leisure centre; and they consolidated the intention to be more physically active by writing down how, when and where physical activity would happen. In this example, APRN1 used the language of change, made it real for the patient, and gave the patient ownership over the planned behaviour change. This is very different from the more traditional language of advice displayed elsewhere (e.g., from Practice Nurse 3: “*You say ‘you’ve got an awful lot of weight to lose’ and I’m too hard [on them]”*) and demonstrates the range of skills required to undertake behaviour change interventions in Primary Care.

Patients are used to the advice-giving dynamic, making this behaviour change component challenging. By talking of *“[taking] it on board”*, Patient 22 perceived the check as an advice-giving opportunity.

*He just said try to cut out snacking and high fat foods. So it’s kind of what you already know but sometimes when somebody’s done your weight and you know, you find out what a healthy BMI is and then what yours is and then because you’re over 40, it does make you, well it made me just think, “right do you know what?”. So I’ve started doing soups and stuff for lunch and things. So I am trying to, you know, however long it lasts, but I am trying to take it on board. (P22)*

Also note the doubt about the longevity of changes made (*“however long it lasts”*). This implies a lack of self-efficacy in making changes which may be avoided by adopting the SMART goal approach. Further problems with advice-giving identified relate to the person giving the advice. Giving advice assumes expertise and ideally a role model. Some participants raised concerns either because they felt hypocritical giving that advice (Practice Nurse 1) or uncomfortable receiving it from someone they thought did not follow it (Patient 9).

*You look at most nurses and doctors, I think you know, they’re really bad examples because we are not the picture of fitness are we? I’m slightly overweight. Some nurses I’ve seen are not good examples. (PN1)*

*I don’t think I would have liked her to say “oh you need to lose another 5 lb because you’re overweight”, this, that and the other because she was hardly Miss Slim herself. (P9)*

### “I should be monitored more”: the need for further follow-up

There was confusion among patients about post-check protocol on two levels: how results of biomedical tests were communicated; and what happened long-term in relation to follow-up and re-testing, despite being informed that they would be retested in five years’ time.

*I should be called back on it really, don’t you think? I should be monitored more. I was a little bit sort of like confused to be honest with you. [..] I mean it’s coming from me. It’s not .. I think, you know, “see you in six months, you should come back and we’ll do another blood test”, you know, it’s me saying, “well perhaps I should be going back now, after six months”. (P2)*

Blood tests needed to be analysed which usually meant off-site in a secondary care laboratory (some alternative providers had their own testing facilities on-site) which required two appointments. Although patients were called back to receive their test results some practices expressed concern that this may result in non-attendance and non-delivery of test results making it impossible to provide patients with their 10 year CVD risk score. Some practices only called in patients found to be at high risk at this second stage and their attendance was not guaranteed (e.g., Practice Manager 4).

*We say to patients to ring back after 10 days. The reason being that some come up healthy, so we won’t get in touch with them so we prefer patients to come in [of their own accord]. But if there is something concerning, the clinician says, “okay, we need to call this patient”, he’ll say to the girls [receptionists] to call the patient. So we’ll invite those patients in. (PM4)*

If practices made decisions about calling patients for follow-up appointments based on the test results then some patients may not get the full benefit from the *personalised advice* and *lifestyle support*. There was support for a results sheet or checklist from both HCPs and patients.

*[They should] get a sheet that all the results can go on and the risk at the bottom. (GP9)*

*I tell you what would have been good, if you were given like a checklist or something with your results on. That would be quite a good thing as a follow-up thing, so you could go home and you say “oh look my cholesterol’s 8.3” or whatever so then you’ve got some information there to work on. (P2)*

Unfortunately in the region evaluated there was no systematic data recording of uptake of *lifestyle support*. However, those involved in provision of lifestyle support or knowledgeable about services available were fairly positive about them but at the same time were sceptical about the longevity of uptake (e.g., Practice Nurse 1 and 4).

*[We] go in a group on a Thursday morning, go for a walk yes. It tends to be females. The response is good during springtime coming into summer and the wintertime the amount of people turning up is quite poor. [..] We’ve got a chemist just across the road, they do the smoking cessation. (PN1)*

*Those that have been have found it very helpful and enjoyed it. But quite often they won’t even go past the referral stage if they don’t feel they want it or they don’t like it. (PN4)*

This meant evaluation of the *lifestyle support* element of the check was limited and no real insights could be drawn from the data.

## Discussion

This evaluation of the NHS Health Check in the HoBtPCT region was conducted to explore HCPs’ and patients’ experiences of it and to examine whether it was delivered according to protocol; to explore patients’ understanding of *personalised advice*; and to determine whether HCPs and patients felt the *lifestyle support* facilitated the adoption of behaviour change in the context of everyday lives. In terms of delivery of the check, there were several inconsistencies, especially in the non-use of SMART goals. This may be related to training; HCPs delivering the check were trained in how to deliver information and use the food box and ‘traffic light’ tape measure but not specifically about the utility of SMART goals and the success of such Behaviour Change Techniques (BCTs) in changing behaviour [6,16].

In relation to patients’ understanding of advice, findings show that assumptions about HCP-patient roles play a part: some patients misunderstood the advice given or lacked the perceived commitment required to make a lasting change. Our results demonstrate that this may be due to *how* advice was provided. Some patients noted dissatisfaction when HCPs did not constitute good role models; advice given from HCPs who were overweight was perceived as inappropriate. Advice depends upon respect from the person giving; by comparison, the ethos of SMART goals is to work collaboratively with patients to give them ownership of goals created. However, some nurses struggled to change their manner of communication with patients and slipped into their ‘traditional’ way of working, i.e., telling patients what do to [17,8]. This suggests different training requirements for different professions or a re-think about the most appropriate professionals to deliver the check.

In terms of patients incorporating *personalised advice* into their everyday lives, we identified problems largely due to the non-use of SMART goals. Setting SMART goals is an ideal introduction for staff to working collaboratively with patients because it requires HCPs to guide patients to identify goals which are detailed and can be incorporated into their daily routine, an essential element of a behavioural intervention [18]. They also increase the likelihood that patients will enact their action plans because they created them. Guidance during and support following goal-setting is crucial for it to be effective [19] and we found referral pathways for *lifestyle support* were patchy at best.

We know from previous research in the UK and overseas that attendance at prevention programmes like the NHS Health Check is affected by a number of factors including patients’ beliefs about health, their perceptions of the role HCPs should have in managing lifestyle, their knowledge of CVD and CVD risk, and their illness perceptions and the connections often made between illness and symptoms [20-23]. It is also clear from a recent review that analyses of effectiveness of prevention programmes like this are problematized by poor fidelity to intervention protocol, heterogeneity of outcome measures, and lack of analytic detail of the behaviour change elements incorporated in the interventions [24].

A clear limitation of this work is its focus within one region of England. However, our findings support those of others within and beyond the UK [7-10]. Furthermore, non-adherence to protocols is a common problem in healthcare interventions, the reversal of which could have a huge impact on their effectiveness [13]. This study did not aim to evaluate the effectiveness of the health check in terms of health or economic outcomes, but the observed lack of consistent recordkeeping would make this impossible. Current evidence questions the clinical effectiveness of general health checks [2,6] but a strength of this research is we know that the use of goal-setting behaviours, such as SMART goals, can lead to initial and maintained behaviour change [4-6,12]. Hence, an emphasis on SMART goals in the *personalised advice* and systematic follow-up of *lifestyle support* take-up would help determine whether they can deliver long-lasting behaviour change that results in lowered risk of CVD.

## Conclusions

The findings presented demonstrate irregularity in the delivery of the NHS Health Check in the region observed and some misconceptions and dissatisfaction among the patients recruited. These results are significant because they illustrate the lost potential to reduce CVD risk through non-compliance to intervention protocol. Addressing this requires investment in HCP training to ensure they understand the rationale of behaviour change elements of public health interventions. This training should extend to Practice managers and others involved in organising the delivery of the health check to ensure appropriate resources are available and to integrate it into standard practice. It was also clear from our results that further work is required to communicate the importance of preventative health to the public and to change attitudes toward preventative medicine. This is essential for the success of prevention programmes in terms of both health and economic outcomes. Further research is required to examine measurable outcomes of the NHS Health Check, but for that to happen there needs to be consistency in process and data collection across regions in the UK where it has been rolled out. Moreover, this qualitative evaluation demonstrates the need to maintain communication between research and practice to ensure we are working together to develop and deliver evidence based public health interventions that are acceptable and feasible to those involved.

## Endnote

^a^Alternative Providers were non-healthcare settings which were recruited to invite patients to attend health checks.

## Abbreviations

APD: Alternative Provider Director^a^
APRN: Alternative Provider Practice Nurse
CCM: Call Centre Manager
CCO: Call Centre Operator
CVD: Cardiovascular Disease
GP: General Practitioner
HCA: Health Care Assistant
HoBtPCT: Heart of Birmingham Teaching Primary Care Trust
MOT: Ministry of Transport motor vehicle roadworthiness test in the UK
PM: Practice Manager
PN: Practice Nurse
NHS: National Health Service in the UK
UK: United Kingdom

## References

[1] NICE: Prevention of Cardiovascular Disease at Population Level. NICE Public Health Guidance 25, 2010 [http://www.nice.org.uk/guidance/ph25]

[2] Putting Prevention First: NHS Health Check: Vascular Risk Assessment and Management Best Practice Guidance. Produced by COI for the Department of Health 2009. [http://www.healthcheck.nhs.uk/document.php?o=224]

[3] McCartney M (2013). Where’s the evidence for NHS Health Checks?. Br Med J.

[4] Darker CD, French DP, Eves FF, Sniehotta FF (2010). An intervention to promote walking amongst the general population based on an ‘extended’ theory of planned behaviour: a waiting list randomised controlled trial. Psychol Health.

[5] Sniehotta FF, Scholtz U, Schwarzer R (2006). Action plans and coping plans for physical exercise: a longitudinal intervention study in cardiac rehabilitation. Br J Health Psychol.

[6] Michie S, Ashford S, Sniehotta FF, Dombrowski SU, Bishop A, French DP (2011). A refined taxonomy of behaviour change techniques to help people change their physical activity and healthy eating behaviours: the CALO-RE taxonomy. Psychol Health.

[7] Troughton J, Jarvis J, Skinner C, Robertson N, Khunti K, Davies M (2008). Waiting for diabetes: perceptions of people with pre-diabetes: a qualitative study. Patient Educ Couns.

[8] Chipchase L, Waterall J, Hill P (2013). Understanding how the NHS Health Check works in practice. Pract Nurs.

[9] Eborall H, Davies R, Kinmonth A-L, Griffin S, Lawton J (2007). Patients’ experiences of screening for type 2 diabetes: prospective qualitative study embedded in the ADDITION (Cambridge) randomized controlled trial. Br Med J.

[10] Emmelin M, Weinehall L, Stenlund H, Wall S, Dahlgren L (2007). To be seen, confirmed and involved – a ten year follow-up of perceived health and cardiovascular risk factors in a Swedish community intervention programme. BMC Public Health.

[11] Eccles MP, Armstrong D, Baker R, Cleary K, Davies H, Davies S, Galsziou P, Ilott I, Kinmonth A-L, Leng G, Logan S, Marteau T, Michie S, Rogers H, Rycroft-Malone J, Sibbald B (2009). An implementation research agenda. Implement Sci.

[12] Drombrowski SU, Sniehotta FF, Avenill A, Johnston M, MacLennan G, Araújo-Soares V (2012). Identifying active ingredients in complex behavioural interventions for obese adults with obesity-related co-morbidities or additional risk factors for co-morbidities: a systematic review. Health Psychol Review.

[13] Bellg AJ, Borrelli B, Resnick B, Hecht J, Minicucci DS, Ory M, Ogedegbe G, Orwig D, Ernst D, Czajkowski S (2004). Enhancing treatment fidelity in health behaviour change studies: best practices and recommendations from the NIH Behavior Change Consortium. Health Psychol.

[14] Robson C (2011). Real World Research: A Resource for Users of Social Research Methods in Applied Settings.

[15] Braun V, Clarke V (2006). Using thematic analysis in psychology. Qual Res Psychol.

[16] Sniehotta FF (2009). Towards a theory of intentional behaviour change: plans, planning, and self-regulation. Br J Health Psychol.

[17] Taylor CA, Shaw RL, Dale J, French DP (2011). Enhancing delivery of health behaviour change interventions in primary care: a meta-synthesis of views and experiences of primary care nurses. Patient Educ Couns.

[18] Borrelli B, Sepinwall D, Ernst D, Bellg AJ, Czajkowski S, Breger R, DeFrancesco C, Levewque C, Sharp DL, Ogedgbe G, Resnick B, Orwig D (2005). A new tool to assess treatment fidelity and evaluation of treatment fidelity across 10 years of health behaviour research. J Consulting Clin Psychol.

[19] Armitage C (2009). Effectiveness of experimenter-provided and self-generated implementation intentions to reduce alcohol consumption in a sample of the general population: a randomized exploratory trial. Health Psychol.

[20] Burgess C, Wright AJ, Forster AS, Dodhia H, Miller J, Fuller F. et al. Influences on individuals’ decisions to take up the offer of a health check: a qualitative study. Health Expect 2014, Available online; doi:10.1111/hex.12212. 10.1111/hex.12212 PMC5810718 24889817

[21] Harkins C, Shaw R, Gillies M, Sloan H, MacIntyre K, Scoular A, Morrison C, MacKay F, Cunningham H, Docherty P, MacIntyre P, Findlay IN (2010). Overcoming barriers to engaging socio-economically disadvantaged populations in CHD primary prevention: a qualitative study. BMC Public Health.

[22] Nielsen K-DB, Dyhr L, Lauritzen T, Malterud K (2004). “You can’t prevent anything anyway” a qualitative study of beliefs and attitudes about refusing health screening in general practice. Fam Pract.

[23] Krogsbøll LT, Jørgensen KJ, Grønhøj Larsen C, Gøtzsche PC (2012). General health checks in adults for reducing morbidity and mortality from disease. Cochrane Database Syst Rev.

[24] Holland C, Cooper Y, Shaw R, Pattison H, Cooke R (2013). Effectiveness and uptake of screening programmes for coronary heart disease and diabetes: a realist review of design components used in interventions. BMJ Open.

